# Editorial

**Published:** 1839-02-16

**Authors:** 


### THE MEDICAL EXAMINER.

PHILADELPHIA, FEB. 16, 1839.

### UNIVERSITY OF THE CITY OF NEW YORK.

This institution is unfortunate enough in some respects. Its council, infected with the same mania for government which caused the resignation of a faculty of arts five years since, has obliged the medical professors to resign in a body. It seems that, for the most part, they are still associated,—and, in all probability, an attempt will be made to form a new medical institution.

The difficulties which took place between the professors and council, arose from a common subject of misunderstanding. The council required from the professors contributions in the form of rent, &c., which would have been exorbitant in an old and flourishing institution. That is, if the University had already been in successful action, and had offered classes of three hundred pupils, it would, to say the least of it, have been highly illiberal to have exacted terms of the nature of those required by the council of the New York University. The question, however, was not one of mere policy,—it was clearly impossible for the professors to accede to the demands of the council, without incurring the risk of very considerable pecuniary obligations.

We know that a medical school which is connected with a literary institution, is pretty sure of being heavily taxed towards the support of the institution in general. When the school is long established, has acquired a name, and may be regarded as a source of permanent income, it will bear such a drain upon its resources. We would not be understood as sanctioning these demands,—they are, to us, clearly unjust,—and it is, in our opinion, entirely at variance with a just administration, to withdraw funds which are derived from a successful medical department of a school, and apply them to the support of the other branches of the institution. It is very obvious that if the professors are able to keep together large classes, and to derive from them a larger income than is necessary for their just remuneration, the excess should be devoted to increasing the means of instruction, to the purchase of apparatus, and preparations of various kinds, which are so necessary to the successful teaching of the medical sciences. Still, there can never be a difficulty in finding competent men to fill the chairs as soon as vacancies occur; for the established standing of the school is, in itself, of actual value, and for a connexion with it every new incumbent is willing to pay a certain proportion of the fees of the students.

In a new institution like the University of New York, no advantage whatever could be derived from a connexion with it, other than the use of the rooms, and the privilege of conferring degrees. The medical professors of the University of New York were perfectly willing, and had already agreed to pay a fair rent for the use of the rooms, but they very properly refused to pay for the privilege of conferring degrees. For, it is quite plain that the demand of the council could never have been founded on any other ground than the fact of the charter having a supposed pecuniary value. The experience of other institutions would have taught the council that the right of conferring degrees is, in itself, of little or no value, until the reputation of the professors, and, consequently, of the school, has drawn together a considerable class of students. At their commencement, medical schools have many difficulties to contend with, and require the fostering aid of those who are invested with the government of the institutions with which they may be connected.

The plan of instruction which was adopted in the University of New York, differed in some respects from that pursued in the older schools. The usual seven professors were appointed, and an attendance on their lectures would entitle the student to a degree upon examination; but an additional number were added to the faculty, as extraordinary professors, to enable those pupils who might desire a more finished education to continue their studies, and pursue a course of instruction nearly as complete as that offered by the continental schools. Whether this innovation would have proved successful, could only have been learned from experience; but, at any rate, the laudable attempt to extend the course of instruction was worthy of a fair trial.

---

*Died*, at Louisville, Kentucky, on the 26th ult., of acute pulmonary consumption, Dr. DANIEL BRENT, in the 22d year of his age, son of Col. Wm. Brent, of Washington.

Dr. Brent graduated at Philadelphia a year since, and is deeply regretted by his friends and associates, as a physician of high promise, and of exemplary private character.

